# Association of Electronic Health Record Vendors With Hospital Financial and Quality Performance: Retrospective Data Analysis

**DOI:** 10.2196/23961

**Published:** 2021-04-14

**Authors:** Bradley Beauvais, Clemens Scott Kruse, Lawrence Fulton, Ramalingam Shanmugam, Zo Ramamonjiarivelo, Matthew Brooks

**Affiliations:** 1 School of Health Administration College of Health Professions Texas State University San Marcos, TX United States

**Keywords:** electronic health records, medical informatics, hospitals, delivery of health care, financial management, quality of health care, treatment outcome

## Abstract

**Background:**

Electronic health records (EHRs) are a central feature of care delivery in acute care hospitals; however, the financial and quality outcomes associated with system performance remain unclear.

**Objective:**

In this study, we aimed to evaluate the association between the top 3 EHR vendors and measures of hospital financial and quality performance.

**Methods:**

This study evaluated 2667 hospitals with Cerner, Epic, or Meditech as their primary EHR and considered their performance with regard to net income, Hospital Value–Based Purchasing Total Performance Score (TPS), and the unweighted subdomains of efficiency and cost reduction; clinical care; patient- and caregiver-centered experience; and patient safety. We hypothesized that there would be a difference among the 3 vendors for each measure.

**Results:**

None of the EHR systems were associated with a statistically significant financial relationship in our study. Epic was positively associated with TPS outcomes (*R^2^*=23.6%; β=.0159, SE 0.0079; *P*=.04) and higher patient perceptions of quality (*R^2^*=29.3%; β=.0292, SE 0.0099; *P*=.003) but was negatively associated with patient safety quality scores (*R^2^*=24.3%; β=−.0221, SE 0.0102; *P*=.03). Cerner and Epic were positively associated with improved efficiency (*R^2^*=31.9%; Cerner: β=.0330, SE 0.0135, *P*=.01; Epic: β=.0465, SE 0.0133, *P*<.001). Finally, all 3 vendors were associated with positive performance in the clinical care domain (Epic: β=.0388, SE 0.0122, *P*=.002; Cerner: β=.0283, SE 0.0124, *P*=.02; Meditech: β=.0273, SE 0.0123, *P*=.03) but with low explanatory power (*R^2^*=4.2%).

**Conclusions:**

The results of this study provide evidence of a difference in clinical outcome performance among the top 3 EHR vendors and may serve as supportive evidence for health care leaders to target future capital investments to improve health care delivery.

## Introduction

### Background

In the first part of the 20th century, health care predominantly revolved around a single health care provider, diagnosing and treating patients within the confines of their office or in the patient’s home, and patient medical histories were recorded on paper. However, as the US health care industry has progressed and modernized, the proliferation of information technology has accelerated. In 2013, US health information technology investment totaled US $2.8 billion; however, by 2017, it had reached a staggering US $7.1 billion [1]. However, some reports indicate that hospitals have struggled to remain profitable during this same time frame [2]. Many health care executives are questioning the return on investment in hospital technology and wondering whether their capital outlay will result in improved financial outcomes [3].

Numerous studies have noted the high expense of care delivery yet low health care information technology proliferation in the United States. Citing the advancement of similar technology in other developed countries, researchers have indicated that through increased investment in health information technology, the United States can lower overall health care spending and simultaneously improve quality of care and patient outcomes [4,5]. As a result, the federal government has been heavily involved in the modernization of health information technology. As early as 2004, President George W Bush called for computerized health records in his State of the Union address and offered a strategy to provide Americans access to electronic health records (EHRs); unfortunately, he did not earn sufficient funding to change provider behavior [6]. In 2009, President Barack Obama signed the Health Information Technology for Economic and Clinical Health (HITECH) Act as part of the American Recovery and Reinvestment Act (ARRA) and originally set aside US $27 billion for an incentive program that encouraged hospitals and providers to adopt EHR systems [7,8]. This legislation prompted the adoption of EHRs in 2014 and established time frames for mandated EHR adoption. Furthermore, with the passage of the Medicare Access and CHIP Reauthorization Act, Medicare introduced the Medicare EHR Incentive Program, and the Merit-Based Incentive Payment System to advance the meaningful use of EHRs. As a result, by 2015, 96% of hospitals and 87% of physician practices implemented EHRs [9]. By mid-2016, the total federal government investment in EHR rose to US $35 billion and continued to rise [10].

One could argue that the proliferation of health care technology spending did not occur organically. The hospital and health care industry shifted to the use of EHR systems primarily because of the financial incentives incorporated within the ARRA and HITECH legislation [11]. HITECH provided eligible professionals who demonstrated the meaningful use of an EHR qualified for payments of US $18,000 in the first year; US $12,000 for the second year; US $8000 for the third year; US $4000 for the fourth year; and US $2000 for the fifth year [12]. An eligible professional was generally considered to be a physician. After 2015, physicians who failed to meaningfully use EHRs were subject to reductions in Medicare and Medicaid reimbursement. Meaningful use of an EHR includes 3 components: (1) the EHR must be certified and include e-prescribing capabilities; (2) the technology must provide for the electronic exchange of personal health information with other EHR systems (interoperability); and (3) the system must produce reports utilizing various clinical and quality metrics.

Beginning in 2011, incentive payments were also available for eligible hospitals that showed meaningful use of EHR and that submitted quality metrics based on criteria identified by the US Department of Health and Human Services. Incentive amounts were phased out in 2015 for hospitals that had not implemented a meaningful EHR. In addition, incentive payments were not available for hospitals that were not meaningful EHR users [13]. Despite the robust startup incentives offered by the federal government and continuing support provided via increased Medicare reimbursement, questions remain if the investment in health care information technology is sustainable. The adoption of a comprehensive EHR system can surpass several billion dollars for a large health care system [14]. The sustainability of these systems can approach several hundred million dollars annually, and numerous health care systems report significant implementation and sustainment cost overages [15,16]. Furthermore, despite the prevalent adoption of EHR systems since the passage of the HITECH Act, sharing of health care data and interoperability of information technology remains to be elusive. There remains to be little financial incentive to share and use data to reduce costs or improve the quality of care [17].

Thus, we seek to assess the association between investment in information technology and the dominant EHR platforms each have on the financial and quality outcomes of hospitals in the United States. We hypothesize that there is a difference in outcomes among the 3 vendors for each measure. Although extensive research has focused on the perceived and actual benefits of information technology in health care, this is an area of research that has not been fully evaluated. The body of literature predating the passage of the HITECH and Affordable Care Acts is fairly robust; however, with the passage of these legislative acts and the rate of change in technology, many of these studies are now outdated, particularly with respect to the direct impact of specific EHR vendors [18-20].

### Literature Review

Two recent studies examined the relationships between health information technology capital expenditure and both financial and quality outcomes and aligned very closely with our work. Wang et al [21] examined the impact of investment in health information technology on hospital financial performance and productivity. In a later study, Wang and Gibbs [22] offered a framework to compare the performance of EHR systems. We intend to build on these studies in a few key areas [21,22].

First, in the 2018 and 2019 studies, the key financial outcome variable examined was return on assets (ROA). Net revenue per staffed bed was also considered in the 2018 study. Broadly speaking, financial performance can be assessed in 4 main areas: (1) profitability or return on investment, (2) liquidity, (3) leverage, and (4) operating efficiency. Within each category, there are several variables to consider. Although ROA and revenue per bed are important, we believe that greater operational clarity can be achieved with a specific focus on the income statement and net income.

Second, quality was evaluated in the 2019 study via the Hospital Value–Based Purchasing (HVBP) Total Performance Score (TPS). These quality measures are all important to the field; however, we view the level of utilization of beds as a suboptimal measure of quality that is not well supported in the literature. The number of times a bed turns over speaks more to the volume of services demanded but offers little insight with respect to the quality of those services. We intend to focus more on the dimensions of HVBP and its subdomains. The contributory factors that are evaluated to produce the TPS include patient perceptions of care and measures of patient safety, process of clinical care delivery, and efficiency and cost reduction.

Third, both the 2018 and 2019 studies included important market characteristic variables as controls. These included hospital size, market concentration index (MCI), payer mix (Medicare and Medicaid; ie, the percentage of revenue coming from each of those programs), uncompensated care cost, ownership (governmental, proprietary, and nonprofit), teaching status, geographic classification, and year fixed effects. We suggest that additional variables may be insightful, as several other factors have been shown to influence both financial and quality outcomes [23-25]. Additional control variables worth considering are the level of outpatient services rendered, urban versus rural location, average length of stay, case mix index, wage index, sole community provider status, system membership, and geographic region [26]. These variables can further clarify the strength of the association between the independent variable of interest and our targeted dependent variables, while also serving to diminish any possible omitted variable bias.

Fourth, the 2019 study considers various EHR systems but does not clearly identify which vendors perform better or worse based on the available data. To the authors’ credit, they did not want the research to be construed as the promotion of a particular vendor. However, in our view, the health care industry is in an era of evidence-based medicine and management. Thus, we believe that it is appropriate to identify the system in question. This more transparent approach, coupled with more specific outcome data that clearly identifies practitioner actionable evidence, should provide greater practical insight and facilitate improved organizational decision making.

In the following sections, we integrate and broaden the investigation of previous research efforts and evaluate the impact of 3 of the largest EHR providers’ performance on measures of finance and quality. Quite simply, we would like to determine which EHR system performs the best and seek to provide health care leaders with an additional evidentiary basis for making EHR adoption decisions. Given the variation in EHR system cost, options, ease of use, training requirements, and on-site and follow-up support, we recognize that this can be a highly complex decision. Although we hypothesize that there is a difference in performance among the 3 EHR vendors in terms of financial and quality performance, we are uncertain a priori where each system will perform the best on the evaluation measures we have selected.

## Methods

### Data and Sample

The data for this study were extracted from two primary sources: the Definitive Health Care database and the American Hospital Association (AHA) Annual Survey database for 2018. The Definitive Health Care database provided the dependent and independent variables of interest, in addition to most of the control variables for this study. The Definitive Health Care database compiles US hospital data sources including Medicare Cost Reports, commercial claims data, Medicare Standard Analytics Files, Centers for Medicare and Medicaid Services (CMS) Hospital Compare, and many other data elements [27]. The cost report contains provider information such as facility characteristics, utilization data, cost and charges by cost center (in total and for Medicare), Medicare settlement data, and financial statement data. The AHA Annual Survey database provided the remaining data for the geographic region control variables [28]. All variables were linked with the 2 contributing data sources based on the Medicare provider number. Data on a total of 2667 short-term acute care hospitals were accumulated for analysis.

### Measures—Dependent Variables

**Figure 1:**
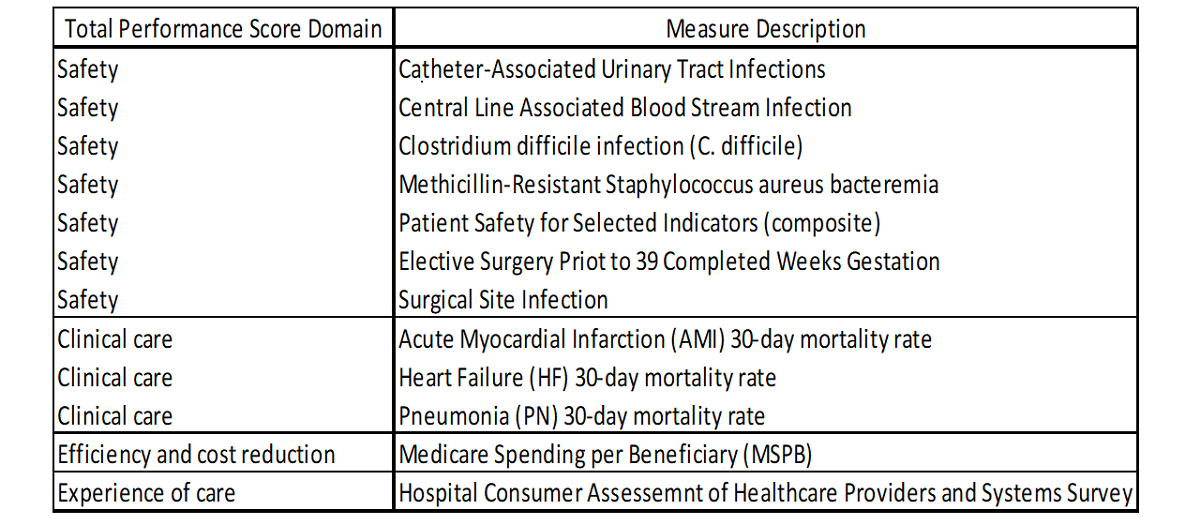
Financial year 2018 Hospital Value–Based Purchasing program measures.

Table 1 shows the full complement of the study variables. Our study included 2 types of dependent variables drawn from the Definitive Health Care data set. The first set of data comes from the hospital income statement: net income scaled in millions of dollars. The second set of dependent variables is drawn from the hospitals’ 2018 value-based purchasing scores and includes (1) the TPS, (2) patient experience score, (3) clinical process score, (4) efficiency score, and (5) the safety score. Each hospital’s TPS is a weighted measure of performance based on each of the other areas listed, while each subordinate measure is an aggregation of several commonly tracked clinical and administrative criteria, as shown in Figure 1.

Under the CMS HVBP Program, Medicare makes incentive payments to hospitals based on how well they perform on each measure compared with other hospitals’ performance during a baseline period and how much they improve their performance on each measure compared with the baseline reporting period [29]. All value-based purchasing variables are unweighted and based on a scale of 0 to 100, with higher scores being better, and have been validated by CMS for validity and reliability [30]. The total number of value-based purchasing participating hospitals provided a size limit to the study. A missingness map using Amelia, a program for missing data developed by Honaker et al [31], revealed approximately 1% missing from 2667 observations and 32 variables (k). The maximum proportion missing from any column was 12.5% and from any row was 10.1%; therefore, these values were conservatively imputed with the median using R Statistical Software [32].

**Table 1:** Variables and operational definitions.

Variable	Original source	Definition
Cerner	Definitive Health Care	Hospital using the Cerner EHR^a^ as its primary EHR platform
Epic	Definitive Health Care	Hospital using the Epic EHR as its primary EHR platform
Meditech	Definitive Health Care	Hospital using the Meditech EHR as its primary EHR platform
TPS^b^	CMS^c^	The TPS is derived from 4 equally weighted domains in financial year 2018: Clinical care Patient experience of care Safety Efficiency and cost reduction
Patient experience score	CMS	Composite of 9 measures extracted from the hospital consumer assessment of health care providers and systems survey
Clinical care score	CMS	Composite of 3 mortality measures: acute myocardial infarction, heart failure, and pneumonia
Efficiency and cost reduction score	CMS	Medicare Spending Per Beneficiary
Safety score	CMS	Composite of 7 safety related rates: catheter-associated urinary tract infections, central line-associated blood stream infection, clostridium difficile infection, methicillin-resistant staphylococcus aureus, patient safety for selected indicators composite, elective delivery before 39 completed weeks gestation, and surgical site infections
For-profit status	Definitive Health Care	Hospitals operated by investor-owned organizations
Number of beds	Definitive Health Care	Number of staffed beds
Rural status	Definitive Health Care	Hospital located in a nonmetropolitan county or a hospital within a metropolitan county that is far away from the urban center, as defined by the Health Resource Services Administration
Government status	Definitive Health Care	Hospitals operated by local, county, or state government
Teaching status	Definitive Health Care	Hospitals affiliated with universities, colleges, medical schools, or nursing schools
Outpatient service mix	Definitive Health Care	Percent of care delivered in an outpatient setting
Average length of stay	Definitive Health Care	The average number of days that patients spend in hospital, measured by dividing the total number of days stayed by all inpatients during a year by the number of admissions or discharges
Case mix	Definitive Health Care	The case mix index is the average relative diagnosis related group weight of a hospital’s inpatient discharges, calculated by summing the Medicare Severity-Diagnosis Related Group weight for each discharge and dividing the total by the number of discharges
Government payer mix	Definitive Health Care	The proportion of hospital reimbursement from governmental sources (Medicare, Medicaid, TRICARE, etc)
Wage Index	Definitive Health Care	A labor market area’s wage index value is the ratio of the area’s average hourly wage to the national average hourly wage
Sole community hospital	Definitive Health Care	A sole community hospital classified by specific criteria from CMS (distance from other like hospitals, rural, travel time, number of beds, etc)
System member	Definitive Health Care	An entity that owns or has owned 2 or more hospitals. In addition, health systems may also maintain ownership of other postacute or ambulatory sites of care
Market concentration	Definitive Health Care	The Herfindahl-Hirschman Index measure of market concentration was used to determine market competitiveness. It is calculated by squaring the market share of each firm competing in a market and then summing the resulting numbers
Average age of facility	Definitive Health Care	Average age of facility is calculated using the accumulated depreciation (total depreciation) and the depreciation expense (depreciation over a single period)
Occupancy rate	Definitive Health Care	Measure of utilization calculated as (inpatient days of care or bed days available)×100
Region	American Hospital Association	Regions of the United States as defined by the American Hospital Association

^a^EHR: electronic health record.

^b^TPS: total performance score.

^c^CMS: Centers for Medicare and Medicaid Services.

### Measures—Independent and Control Variables

Our independent variables of interest included the top EHR systems used by hospitals in the United States (ie, Cerner, Epic, Meditech, or other), as reported in the Definitive Health Care database. These variables were included in our analysis as a dichotomous variable for each EHR system of interest (“1” if the system was used, “0” if not). Consistent with previous research, we also included several organizational-level control variables to account for other explanatory factors that could influence financial and quality outcomes. These variables included for-profit ownership status, number of beds, rural or urban geographic location, government ownership, teaching status, outpatient service mix, average length of stay, case mix, government payer percentage, wage index, sole community provider designation, system membership, MCI, occupancy rate, and geographic location by the AHA region. All analyses were performed using collinearity diagnostics. The variance inflation factor exceeded 10 in our analyses. Table 1 shows the data sources and operational definitions of the variables used in this study.

## Results

### Overview

Descriptive statistics and pairwise correlations were also calculated. The distributions of most of the dependent variables (income, TPS, experience score, clinical score, and safety) were relatively normal; however, the HVBP efficiency score was skewed to the right. Box-Cox analysis of the variable suggested a negative square root transformation, but for interpretability, it was not transformed. All dependent variables were min-max scaled between 0 and 1 for easier interpretation as percentile scores (eg, income percentile). All statistical analyses were performed using the R Statistical Software [32]. In all the analyses, a two-tailed *P* value <.05 was considered statistically significant.

Table 2 provides detailed descriptive statistics for each variable. Participating hospitals had a mean net income of US $15.01 million (SD US $109.04 million); TPS mean of 37.26 (SD 11.16); patient experience mean of 33.36 (SD 18.07); clinical process score mean of 59.45 (SD 19.24); efficiency score mean of 19.39 (SD 24.75); and safety score mean of 53.01 (SD 17.74). In all cases, higher scores on the HVBP variables were better. EHR vendors are represented in the following proportions in our sample: Epic: 39.85% (1063/2667; SD 0.48); Cerner: 23.39% (624/2667; SD 0.42); Meditech: 19.98% (533/2667; SD 0.39); and “other”: 16.78% (447/2667; SD 0.39).

Among the numerous organizational characteristics included in our analysis as control variables, we observe 18.00% (480/2667) of the hospitals in the study population are for-profit facilities (SD 0.38), 22.98% (613/2667) are in rural locations (SD 0.42), 45.97% (1226/2667) are teaching facilities (SD 0.49), 72.97% (1946/2667) are affiliated with a health care system (SD 0.45), and that the hospitals are widely distributed across each of the AHA geographic regions.

**Table 2:** Descriptive statistics.

Variable	Values, mean (SD)
Net income (in millions; US $)	15.01 (109.04)
Total performance score	37.26 (11.17)
Patient experience score (unweighted)	33.36 (18.07)
Clinical process score (unweighted)	59.45 (19.24)
Efficiency score (unweighted)	19.39 (24.75)
Safety score (unweighted)	53.01 (17.74)
EHR^a^-Cerner	0.23 (0.42)
EHR-Epic	0.40 (0.48)
EHR-Meditech	0.20 (0.40)
EHR-other	0.17 (0.39)
For-profit	0.18 (0.40)
Beds	214.75 (185.47)
Rural	0.23 (0.42)
Government	0.13 (0.34)
Teaching	0.47 (0.50)
Outpatient service mix	0.53 (0.15)
Average length of stay	4.30 (0.92)
Case mix index	1.61 (0.28)
Government payer percent	0.71 (0.11)
Wage index	1.00 (0.20)
Sole community provider	0.08 (0.28)
System member	0.73 (0.45)
Market concentration index	0.34 (0.330)
Occupancy rate	0.57 (0.17)
Average age of facility	12.95 (9.23)
Region 1^b^ (Connecticut, Maine, New Hampshire, Rhode Island, and Vermont)	0.04 (0.20)
Region 2 (New Jersey, New York, and Pennsylvania)	0.12 (0.32)
Region 3 (Delaware, Kentucky, Maryland, North Carolina, Virginia, West Virginia, and Washington, DC)	0.08 (0.28)
Region 4 (Alabama, Florida, Georgia, Mississippi, South Carolina, Tennessee, and Puerto Rico)	0.17 (0.37)
Region 5 (Illinois, Michigan, Indiana, Ohio, and Wisconsin)	0.17 (0.37)
Region 6 (Iowa, Kansas, Minnesota, Missouri, Nebraska, North Dakota, South Dakota)	0.08 (0.27)
Region 7 (Arkansas, Louisiana, and Texas)	0.13 (0.35)
Region 8 (Arizona, Colorado, Idaho, Montana, New Mexico, Utah, and Wyoming)	0.07 (0.260)
Region 9 (Alaska, California, Hawaii, Nevada, Oregon, and Washington)	0.13 (0.34)

^a^EHR: electronic health record.

^b^The representative geographical region is American Hospital Association Region 1 (Connecticut, Maine, New Hampshire, Rhode Island, and Vermont).

### Net Income

Table 3 reflects the results of our regression analyses of hospitals’ utilization of the top 3 EHR vendors and the associated hospital financial performance as measured by net income. On the basis of our analysis of net income regressed on EHR vendors (*R^2^*=10.6%), we see no significant results for any of the vendors, when compared with facilities that fall into the “other” category. Thus, we can say, on average, and when controlling for the numerous organizational factors as controls, none of the EHRs are associated with favorable or unfavorable financial outcomes as measured by net income.

Table 3 also shows additional significant variables in our analysis that are associated with hospital net income, including the number of hospital beds (β=.0001, SE 0.0000; *P*<.001), hospital case mix (β=.0067, SE 0.0032; *P*=.04), hospital wage index (β=−.0200, SE 0.0059; *P*<.001), and several geographic variables. These findings indicate that with a point increase in hospital case mix, net income increases by 0.67%, and with each point increase in the hospital wage index, net income falls by 2%.

**Table 3:** Analysis results for net income and total performance score.

Variable	Net income (adjusted *R*^*2*^=10.64%)				Total performance score (adjusted *R*^*2*^=23.61%)		
	β	SE	Significance (*P* value)	β		SE	Significance (*P* value)
Intercept	.5591	0.0139	<.001	.4027		0.0487	<.001
Cerner	.0007	0.0023	—^a^	−.0021		0.0080	—
Epic	−.0024	0.0023	—	.0159		0.0079	.04
Meditech	.0018	0.0023	—	.0117		0.0079	—
For-profit	.0011	0.0021	—	−.0176		0.6203	.02
Beds	.0001	0.0000	<.001	−.0045		0.0019	<.001
Rural	−.0012	0.0024	—	.0556		0.0084	<.001
Government	−.0017	0.0022	—	−.0125		0.0076	—
Teaching	.0051	0.0016	—	.0129		0.0055	.02
Outpatient service mix	−.0092	0.0073	—	.2137		0.0254	<.001
Average length of stay	−.0015	0.0009	—	−.0215		0.0033	<.001
Case mix	.0067	0.0032	.04	.0254		0.0111	.02
Government payer percent	−.0017	0.0022	—	−.0125		0.0076	—
Wage index	−.0200	0.0059	<.001	.0523		0.0204	.01
Sole community provider	.0044	0.0027	—	.0345		0.0095	<.001
System member	.0002	0.0018	—	.0061		0.0061	—
Market concentration	.0014	0.0028	—	−.0380		0.0098	<.001
Average age of facility	.0000	0.0001	—	.0000		0.0003	—
Occupancy rate	.0042	0.0053	—	.0042		0.0183	—
Region 2^b^ (New Jersey, New York, and Pennsylvania)	−.0071	0.0039	—	−.0166		0.0135	—
Region 3 (Delaware, Kentucky, Maryland, North Carolina, Virginia, West Virginia, and Washington, DC)	−.0077	0.0043	—	.0070		0.0151	—
Region 4 (Alabama, Florida, Georgia, Mississippi, South Carolina, Tennessee, and Puerto Rico)	−.0129	0.0042	<.01	−.0211		0.0146	—
Region 5 (Illinois, Michigan, Indiana, Ohio, and Wisconsin)	−.0060	0.0039	—	.0044		0.0135	—
Region 6 (Iowa, Kansas, Minnesota, Missouri, Nebraska, North Dakota, and South Dakota)	−.0086	0.0044	.05	.0200		0.0152	—
Region 7 (Arkansas, Louisiana, and Texas)	−.0132	0.0043	<.01	−.0187		0.0150	—
Region 8 (Arizona, Colorado, Idaho, Montana, New Mexico, Utah, and Wyoming)	−.0057	0.0045	—	−.0138		0.0155	—
Region 9 (Alaska, California, Hawaii, Nevada, Oregon, and Washington)	−.0218	0.0040	<.001	.0167		0.0140	—

^a^Not significant.

^b^Referent geographical region is Region 1 (Connecticut, Maine, New Hampshire, Rhode Island, and Vermont); referent electronic health record is “other.”

### Total Performance Score

Table 3 also shows the results of our regression analyses of the association of EHRs with HVBP quality measures. On the basis of our analysis of TPS regressed on EHR vendors (*R^2^*=23.6%), Epic reflects only statistically significant results (β=.0159, SE 0.0079; *P*=.04). These results indicate that the Epic EHR is associated with a 1.6% higher performance score when compared with facilities that fall into the “other” category. Neither Meditech nor Cerner were associated with significant results.

Table 3 further indicates several control variables that are significantly associated with TPS performance score. These variables include for-profit ownership (β=−.0176, SE 0.6203; *P*=.02), number of hospital beds (β=−.0045, SE 0.0019; *P*<.001), rural designation (β=.0556, SE 0.0084; *P*<.001), teaching designation (β=.0129, SE 0.0055; *P*=.02), outpatient service mix (β=.2137, SE 0.0254; *P*<.001), average length of stay (β=−.0215, SE 0.0033; *P*<.001), case mix (β=.0254, SE 0.0111; *P*=.02), wage index (β=.0523, SE 0.0204; *P*=.01), sole community provider designation (β=.0345, SE 0.0095; *P*<.001), and the MCI (β=−.0380, SE 0.0098; *P*<.001).

Among other findings, these results imply, on average, for-profit facilities perform 1.7% lower on the TPS measure. In addition, with each day increase in average length of stay, we observed a 2.2% decrease in TPS, and with each point increase in market concentration, we see an associated 3.8% decrease in TPS performance. However, with each point increase in the case mix index, wage index, and outpatient service mix, we observed a 2.5%, 5.2%, and 21.4% increase in TPS outcomes, respectively. Teaching hospitals are also associated with a 1.2% higher level of performance than nonteaching facilities.

### Efficiency Score

In Table 4, we also show the evaluation of efficiency performance scores regressed on EHR vendors (*R^2^*=31.9%). Cerner (β=.0330, SE 0.0135; *P*=.01) and Epic (β=.0465, SE 0.0133; *P*<.001) were positively associated with improved efficiency quality scores approximately 3.3% higher (Cerner) and 4.7% higher (Epic) than hospitals in the “other” category. Meditech was not associated with any significant results.

Table 4 also indicates several variables that are significantly associated with hospital efficiency. These variables include the number of hospital beds (β=−.0001, SE 0.0000; *P*=.002), rural designation (β=.0723, SE 0.0142; *P*<.001), outpatient service mix (β=.4831, SE 0.0429; *P*<.001), average length of stay (β=−.0233, SE 0.0055; *P*<.001), case mix (β=−.0698, SE 0.0188; *P*<.001), government payer percent (β=−.2486, SE 0.0375; *P*<.001), wage index (β=.1145, SE 0.0344; *P*<.001), sole community provider designation (β=.0888, SE 0.0160; *P*<.001), occupancy rate (β=.0656, SE 0.0310; *P*=.03), and several regional variables. This implies, on average, there is a statistically significant 2.3% decrease in efficiency score with each day increase in average length of stay, a 6.9% decrease with each point increase in the case mix index, and a 24.9% decrease in efficiency is associated with each point increase in government payer percentage.

**Table 4:** Analysis results for efficiency score and patient experience score.

Variable	Efficiency score (adjusted *R*^*2*^=31.98%)				Patient experience score (adjusted *R*^*2*^=29.3%)			
	β	SE	Significance (*P* value)	β		SE	Significance (*P* value)	
Intercept	.0012	0.0822	—^a^	.3441		0.0612	<.001	
Cerner	.0330	0.0135	.01	−.0002		0.0100	—	
Epic	.0465	0.0133	<.001	.0292		0.0099	.003	
Meditech	.0161	0.0133	—	.0157		0.0099	—	
For-Profit	−.0119	0.0125	—	−.0542		0.0093	<.001	
Beds	−.0001	0.0000	.002	−.0001		0.0000	<.001	
Rural	.0723	0.0142	<.001	.0517		0.0105	<.001	
Government	−.0065	−0.0065	—	.0044		0.0095	—	
Teaching	−.0050	0.0093	—	.0270		0.0069	<.001	
Outpatient service mix	.4831	0.0429	<.001	.3091		0.0320	<.001	
Average length of stay	−.0233	0.0055	<.001	−.0251		0.0041	<.001	
Case mix	−.0698	0.0188	<.001	.0100		0.0140	<.001	
Government payer percent	−.2486	0.0375	<.001	.0044		0.0095	<.001	
Wage index	.1145	0.0344	<.001	−.0744		0.0256	—	
Sole community provider	.0888	0.0160	<.001	.0081		0.0119	—	
System member	.0138	0.0103	—	−.0124		0.0077	—	
Market concentration	.0158	0.0165	—	−.0586		0.0123	<.001	
Average age of facility	.0004	0.0005	—	.0004		0.0003	—	
Occupancy rate	.0656	0.0310	.03	−.0618		0.0230	.007	
Region 2^b^ (New Jersey, New York, and Pennsylvania)	.1062	0.0227	<.001	−.0649		0.0169	<.001	
Region 3 (Delaware, Kentucky, Maryland, North Carolina, Virginia, West Virginia, and Washington, DC)	.1627	0.0254	<.001	−.0522		0.0189	.005	
Region 4 (Alabama, Florida, Georgia, Mississippi, South Carolina, Tennessee, and Puerto Rico)	.0949	0.0247	<.001	−.0255		0.0184	—	
Region 5 (Illinois, Michigan, Indiana, Ohio, and Wisconsin)	.0708	0.0228	.002	−.0254		0.0170	—	
Region 6 (Iowa, Kansas, Minnesota, Missouri, Nebraska, North Dakota, and South Dakota)	.2219	0.0257	<.001	−.0472		0.0191	.01	
Region 7 (Arkansas, Louisiana, and Texas)	.0481	0.0254	—	−.0066		0.0189	—	
Region 8 (Arizona, Colorado, Idaho, Montana, New Mexico, Utah, and Wyoming)	.1702	0.0262	<.001	−.0977		0.0195	<.001	
Region 9 (Alaska, California, Hawaii, Nevada, Oregon, and Washington)	.2693	0.0237	<.001	−.0740		0.0176	<.001	

^a^Not significant.

^b^Referent geographical region is Region 1 (Connecticut, Maine, New Hampshire, Rhode Island, and Vermont); referent electronic health record is “other.”

### Patient Experience Score

Table 4 provides the final analysis results of our evaluation of hospitals’ patient experience performance scores regressed on EHR vendors. Epic was positively associated with higher patient perceptions of quality scores 2.9% higher than hospitals in the “other” category (*R^2^*=29.3%; β=.0292, SE 0.0099; *P*=.003).

Table 4 provides additional insight pertaining to the significant association between the control variables included in our study and patient experience scores. These variables include for-profit ownership (β=−.0542, SE 0.0093; *P*<.001), number of beds (β=−.0001, SE 0.0000; *P*<.001), rural status (β=.0517, SE 0.0105; *P*<.001), teaching (β=.0270, SE 0.0069; *P*<.001), outpatient service mix (β=.3091, SE 0.0320; *P*<.001), average length of stay (β=−.0251, SE 0.0041; *P*<.001), case mix (β=.0100, SE 0.0140; *P*<.001), government payer percent (β=.0044, SE 0.0095; *P*<.001), market concentration (β=−.0586, SE 0.0123; *P*<.001), occupancy rate (β=−.0618, SE 0.0230; *P*=.007), and several geographic regions.

These results imply that, on average, the for-profit hospitals in our study scored 5.4% lower on the HVBP patient experience scores. In addition, for each additional day in the hospital, the patient experience scores decreased by 2.5%. Each point increase in market concentration and occupancy rate also reduces patient experience by 5.9% and 6.2%, respectively. Conversely, rural and teaching hospitals are associated with higher patient experience, with associated increased scores of 5.2% and 2.7%, respectively.

### Patient Safety Score

Table 5 provides insight into our research on patient safety performance scores regressed on EHR vendors (*R^2^*=24.3%). Epic (β=−.0221, SE 0.0102; *P*=.03) was negatively associated with patient safety quality scores of 2.2% lower than hospitals in the “other” category. Meditech and Cerner scores were not associated with significant results.

Table 5 also provides details regarding the significant associations between the control variables included in our study and patient safety scores. These variables include the number of hospital beds (β=−.0002, SE 0.0000; *P*<.001), rural status (β=.0420, SE 0.0109; *P*<.001), teaching (β=.0168, SE 0.0071; *P*=.02), outpatient service mix (β=.0965, SE 0.0330; *P*=.003), average length of stay (β=−.0143, SE 0.0042; *P*<.001), case mix (β=−.0812, SE 0.0144; *P*<.001), government payer percent (β=.0746, SE 0.0288; *P*=.009), and occupancy rate (β=−.0919, SE 0.0238; *P*<.001). These results indicate that patient safety is negatively impacted by 1.4% for every day increase in average length of stay and also declines by 8.1% for every point increase in the case mix index. Furthermore, with each percent increase in the hospital occupancy rate, patient safety scores declined by 9.2%. Conversely, patient safety scores were positively associated with rural and teaching hospitals by 4.2% and 1.7%, respectively. In addition, with each percentage increase in government payments, patient safety scores improved by 7.5%.

**Table 5:** Analysis results for patient safety score and clinical process score.

Variable	Patient safety score (adjusted *R*^*2*^=24.35%)					Clinical process score (adjusted *R*^*2*^=4.19%)				
	β	SE	Significance (*P* value)		β		SE		Significance (*P* value)	
Intercept	.8251	0.0632	<.001		.5974		0.0758		<.001	
Cerner	−.0182	0.0104	—^a^		.0284		0.0124		.02	
Epic	−.0221	0.0102	.03		.0389		0.0122		.002	
Meditech	−.0004	0.0103	—		.0274		0.0123		.03	
For-profit	.0022	0.0096	—		.0512		0.0116		<.001	
Beds	−.0002	0.0000	<.001		−.0001		0.0000		.02	
Rural	.0420	0.0109	<.001		.0028		0.0131		—	
Government	−.0083	0.0098	—		−.0264		0.0118		.03	
Teaching	.0168	0.0071	.02		.0167		0.0086		.05	
Outpatient service mix	.0965	0.0330	.003		.0182		0.0396		—	
Average length of stay	−.0143	0.0042	<.001		−.0094		0.0051		—	
Case mix	−.0812	0.0144	<.001		.0223		0.0173		—	
Government payer percent	.0746	0.0288	.009		−.0839		0.0346		.02	
Wage index	−.0314	0.0264	—		.0013		0.0317		—	
Sole community provider	.0237	0.0123	—		.0146		0.0148		—	
System member	.0082	0.0079	—		.0329		0.0095		<.001	
Market concentration	−.0174	0.0127	—		.0323		0.0152		.03	
Average age of facility	−.0003	0.0004	—		.0009		0.0004		.04	
Occupancy rate	−.0919	0.0238	<.001		−.0057		0.0286		—	
Region 2^b^ (New Jersey, New York, and Pennsylvania)	.0051	0.0174	—		−.0014		0.0210		—	
Region 3 (Delaware, Kentucky, Maryland, North Carolina, Virginia, West Virginia, and Washington, DC)	.0150	0.0196	—		.0212		0.0235		—	
Region 4 (Alabama, Florida, Georgia, Mississippi, South Carolina, Tennessee, and Puerto Rico)	−.0056	0.0190	—		.0156		0.0228		—	
Region 5 (Illinois, Michigan, Indiana, Ohio, and Wisconsin)	.0200	0.0175	—		.0275		0.0210		—	
Region 6 (Iowa, Kansas, Minnesota, Missouri, Nebraska, North Dakota, and South Dakota)	−.0013	0.0197	—		−.0171		0.0237		—	
Region 7 (Arkansas, Louisiana, and Texas)	.0055	0.0195	—		−.0126		0.0234		—	
Region 8 (Arizona, Colorado, Idaho, Montana, New Mexico, Utah, and Wyoming)	−.0023	0.0201	—		−.0457		0.0242		—	
Region 9 (Alaska, California, Hawaii, Nevada, Oregon, and Washington)	.0142	0.0182	—		.0181		0.0219		—	

^a^Not significant.

^b^Referent geographical region is Region 1 (Connecticut, Maine, New Hampshire, Rhode Island, and Vermont); referent electronic health record is “other.”

### Clinical Care Performance Score

Finally, Table 5 shows the results of our evaluation of clinical care performance scores regressed on EHR vendors. All 3 vendors were associated with positive performance with Epic (β=.0388, SE 0.0122; *P*=.002), Cerner (β=.0283, SE 0.0124; *P*=.02), and Meditech (β=.0273, SE 0.0123; *P*=.03), reflecting positively associated higher clinical care performance scores between 2.7% (Meditech) and 3.8% (Epic) higher than hospitals in the “other” category. However, on this dependent variable, we recognize that the explanatory power of the regressors is very low (*R^2^*=4.2%).

Table 5 also provides insight into the association between the control variables in our study and clinical process outcomes. The statistically significant variables in our analysis included for-profit status (β=.0512, SE 0.0116; *P*<.001), number of hospital beds (β=−.0001, SE 0.0000; *P*=.02), government operated (β=−.0264, SE 0.0118; *P*=.03), teaching (β=.0167, SE 0.0086; *P*=.05), government payer percentage (β=−.0839, SE 0.0346; *P*=.02), system membership (β=.0329, SE 0.0095; *P*<.001), market concentration (β=.0323, SE 0.0152; *P*=.03), and the average age of the facility (β=.0009, SE 0.0004; *P*=.04). These results indicate that government-operated hospitals are associated with 2.6% lower clinical process scores, and for each point increase in government payer percentage, there is also an 8.3% lower score. However, for-profit, teaching, and system-owned hospitals appear to perform better on this measure by 5.1%, 1.6%, and 3.3%, respectively. Hospitals in concentrated markets also appear to perform better than those in less concentrated markets. With each point increase in market concentration, we observed an increase of 3.2%.

## Discussion

### Principal Findings

In general, our findings were insightful regarding the performance of individual EHR vendors. We did not expect to see a clearly obvious choice of EHR vendor with respect to performance as defined by our financial and quality-focused dependent variables. To this end, we did not ascertain that there is a single EHR that outperforms all other competitors across all of our study measures.

Our findings pertaining to financial outcomes were somewhat interesting in that no single EHR demonstrated a significant and positive association with net income. In most instances, the capital allocation process is predicated on reasonable assurance that there will be some tangible return on investment over a reasonable amount of time. As EHR adoption is a major capital investment and requires a major organizational change and extensive training, some have argued that it may take the organization a few years to see its effect on financial performance. For instance, Collum et al [20] found a statistically significant improvement in the total margin 2 years after EHR adoption in hospitals. However, the authors attributed the observed effect more to HITECH Act incentive payments than operational improvements, primarily because the authors found no significant association with operating margin. Thus, these previous authors’ observations, coupled with our own, continue to indicate that EHR return on investment remains an unsettled matter.

In our evaluation of TPS as a dependent variable, we note that Epic was the singular EHR with a positive and significant association with improved scoring. Given that the TPS is a composite of the other variables in our analysis, it prompted us to examine each of the subdomains’ performance scores more closely. Although we did not see a positive association for Epic in improved financial performance, this EHR recorded positive and significant associations in clinical care, patient experience, and efficiency scoring. Thus, when considered together, this combination of scores across these 3 quality subdomains appears to provide a performance advantage to Epic and a positive association with TPS scoring.

However, Epic also demonstrated a statistically significant and negative association with patient safety, which was not observed in our other vendors’ performance. Although we did not expect to observe a significant and negative association between any EHR vendors and patient safety scoring, upon further research on this topic, we note that our findings appear to be consistent with several previous researchers’ results. Bowman [33] captures these areas of concern very well in her synthesis of 64 studies and papers highlighting numerous areas where EHRs can impose undue burdens on health care providers and introduce the possibility of errors. These include the potential for recording erroneous data entry leading to patient safety hazards, system design flaws, improper system use, inappropriate document capture, erroneous application of copy and paste functions within the medical record, rigid application of prepopulated templates, and errors related to clinical decision support systems such as alert fatigue [33]. In recent years, others have pointed to potential problems with EHRs as a vector for increased risk to patient safety with respect to incorrect use [34,35], malfunctions [36,37], interoperability or system interaction [38], and health information technology blackouts or downtime [36,39]. On the basis of our findings, we can reasonably assume that many of these issues persist.

Our research group discussed the possible reasons for these results. In many ways, our results are supported by independent research. The top vendors that we studied are often highlighted in the KLAS, LLC Research for best in class, most user friendly, and holding buyers’ attention [40]. It is a user-friendly variable that could contribute to the software’s success in efficiency measures. It is possible that all 3 vendors are equally capable of achieving similar efficiency scores, but the fact that users are more familiar with their function and more willing to explore beyond the basic user training that renders Epic more effective in the areas of TPS. One could reasonably assume that the number of years since the hospital adopted the EHR system, and also the stage of adoption, might have had an effect. Hospitals that have adopted the EHR over several years may be more efficient than hospitals that only recently adopted or changed their EHR system. This could have an impact on levels of customer service, capability to integrate modules together, onboarding processes relating to the EHR, and the organization’s capacity to facilitate initial and ongoing training.

Finally, another factor contributing to the success of one vendor over the others could be ownership. Epic and Meditech have proudly and defiantly maintained their private status. This factor could make a vendor more agile in its software development life cycle, enabling them to customize to order or correct flaws rapidly.

### Practice Implications

Those involved in purchasing decisions surrounding EHR should carefully consider areas of focus in the facility, capital expenditure cycles, strategic direction, and willingness of the organization to change EHR vendors. The significant decision to switch EHR vendors is a complex process, and many factors need to be aligned to set up the organization for success. Our research may provide an evidentiary basis for vendor selection. For example, an organization that struggles with improving clinical care performance might consider Epic or Meditech with an understanding that there are numerous other factors that might influence outcomes. A similar choice might be considered for facilities desiring to improve patient experience. However, if patient experience or efficiency—in terms of Medicare Spending Per Beneficiary—is an area of weakness, Epic might be a preferred choice. Ultimately, an organization using a vendor other than these 3 might look at the features that these vendors offer that their vendor does not. Is there something their current vendor could offer and increase measures of efficiency? Regrettably, our research does not extend to the module level of the EHR, so we cannot make any recommendations in that regard.

### Limitations and Recommendations for Future Research

Our study had several limitations. First, this is a single year of data drawn from the 2018 data pertaining to performance within only short-term acute care HVBP participating facilities. Future studies should consider examining the growth or decline of EHR influence on these outcomes over time. Furthermore, as a single-year study, our analysis also does not account for any changes in EHR systems during the delay between baseline and performance reporting periods from which HVBP scores were determined, nor do we include the length of time the EHR has been in place within the hospitals studied. A more in-depth paired analysis could be considered to match the EHR system with the exact time frame of performance. Additional financial and quality outcome variables might also be considered, which could broaden the study unit of observations beyond the HVBP constraint. Finally, as more granular data becomes available pertaining to the specific modules in use at the hospital level, future studies might examine how specific module use is associated with specific clinical outcomes.

Beyond the extensive implementation of EHRs, health care providers, hospitals, and health care facilities invest heavily in other forms of information technology to provide and enhance care delivery. As the industry progresses toward value-based care, organizations are increasingly investing in imaging, telehealth, precision medicine, artificial intelligence, cloud-based computing or data storage, consumer-facing technologies, and disease management technologies. Furthermore, in the days since the start of the COVID-19 pandemic, telemedicine has been showcased as an indispensable capability of the EHR. Another aspect that should be evaluated in the future is the telemedicine capabilities of these vendors. Future research should carefully examine care delivered through this modality and compare the outcomes across the top vendors.

### Conclusions

The return on investment and outcomes associated with EHRs have been a topic of intense focus and debate over the past 2 decades. Up to this point, a research gap has persisted pertaining to the study and transparent disclosure of comparative studies of major EHR vendors. In our analysis of the big 3 vendors—Epic, Cerner, and Meditech—we endeavored to fill that gap. Yet, we can see that clearly answering which system performs the best is complex. The implementation of any of these products can take years, and success is not guaranteed. However, our findings may provide some clarity to health care leaders seeking to develop an evidence base to support future capital investment in EHR systems. If an organization is already considering a switch to a new EHR vendor, the organization can devote sufficient funding for such an undertaking, and if the leadership is willing to lead such a large organizational change, then our study may provide some points of clarity pertaining to the big 3 vendors that might be apt for consideration.

## Abbreviations

AHA: American Hospital Association
ARRA: American Recovery and Reinvestment Act
CMS: Centers for Medicare and Medicaid Services
EHR: electronic health record
HITECH: Health Information Technology for Economic and Clinical Health
HVBP: Hospital Value–Based Purchasing
MCI: market concentration index
ROA: return on assets
TPS: total performance score
